# Automated segmentation of lymph node metastases in colorectal cancer based on convolutional KAN

**DOI:** 10.1515/med-2026-1469

**Published:** 2026-09-07

**Authors:** Peng Wang, Zixiong Fan, Haoyu Wu, Kaijiao Wang, Jinyi Lu, Yang Zhou, Yang Liu, Baoshun Zhang

**Affiliations:** Artificial Intelligence Energy Research Institute, Northeast Petroleum University, Daqing, Heilongjiang, China; Heilongjiang Provincial Key Laboratory of Networking and Intelligent Control, Northeast Petroleum University, Daqing, Heilongjiang, China; Admissions and Employment Office, Northeast Petroleum University, Daqing, Heilongjiang, China; SANYA Offshore Oil & Gas Research lnstitute, Northeast Petroleum University, Sanyan, Hainan, China; Department of Radiology, Harbin Medical University Cancer Hospital, Harbin, Heilongjiang, China; Colorectal Surgery Department, Longnan Hospital of Daqing, Daqing, Heilongjiang, China

**Keywords:** segmentation, colorectal cancer lymph node metastasis, UCKAN, GSMA

## Abstract

Accurate preoperative segmentation of colorectal cancer lymph node metastasis holds significant clinical value for formulating individualized surgical strategies. However, accurate segmentation remains a formidable challenge due to the complex backgrounds, small sizes, irregular shapes, and indistinct boundaries typically exhibited by colorectal cancer lymph nodes. Consequently, this paper proposes the U-Convolutional KANs Attention Net (UCKAN). First, in complex environment, we replace the convolutional layers with the convolutional KAN module, leveraging the learnable non-linear fitting advantage of KAN to enhance the ability to accurately extract the features of small targets. Second, we invented the Global Spatial Multi-scale Attention (GSMA) module, which integrates global context and multi-scale features to improve the model’s sensitivity to small-volume lymph nodes, achieving precise spatial focusing on lesions. Evaluations on practical medical imaging tasks show that the UCKAN model has superior performance., with Dice and IoU scores reaching 91.52 and 83.29 %, respectively, comprehensively outperforming eight advanced medical image segmentation models. The innovative mechanisms proposed in this study effectively address the segmentation challenges associated with low-contrast and multi-scale lesions, providing a reliable quantitative basis for formulating individualized preoperative surgical strategies and demonstrating significant potential for clinical translation.

## Introduction

Accurate dissection of lymph node is a cornerstone of curative resection for colorectal cancer [1]. Numerous clinical studies have demonstrated that lymph node metastasis of colorectal cancer is one of the most critical prognostic factors influencing postoperative recurrence [2], [3], [4]. Colorectal cancer lymph node metastasis (CRC LNM) could result in increasing the 5-year recurrence rate to approximately 30–50 % [5] and directly influences treatment decisions and overall prognosis [2], 6]. Consequently, accurately identifying and delineating suspicious lymph nodes on preoperative MRI (Magnetic Resonance Imaging) holds significant clinical value for developing individualized surgical strategies.

Currently, the incidence rate of rectal cancer ranks third among all types of cancers in worldwide (9.6 %), and it ranks second among diseases causing death due to cancer [7],thereby becoming a serious burden on public health [8]. MRI is the primary imaging modality that provides essential structural and anatomical information for the preoperative assessment of colorectal cancer. Therefore, accurate segmentation of CRC LNM on MRI plays a crucial role in formulating targeted surgical strategies preoperatively [9]. However, in actual clinical scenarios, CRC LNM on MRI usually presents complex surrounding tissues, very small sizes (<5–6 mm), irregular morphologies, and poorly defined boundaries, thereby, making accurate MRI-based segmentation of CRC LNM a highly challenging task [10].

In current clinical practice, preoperative lymph node assessment highly depends on experience of radiologists, slice-by-slice inspection and subjective interpretation. However, due to the limitations of the doctor’s experience level, fatigue, the external environment, and the complexity of the imaging itself, misdiagnosis (23–34 %) and missed diagnosis (18–29 %) are prone to occur in actual diagnoses [11], thereby affecting clinical decisions, treatment strategy selection, surgical scope, and prognosis judgment. Therefore, developing automated segmentation algorithms for colorectal cancer lymph node metastasis could provide clinicians with objective, quantitative assessment support, enhance diagnostic accuracy, reduce workload, and improve the consistency and reliability of the diagnostic workflow.

Due to the recent rapid evolution of deep learning, it has achieved remarkable success in the area of medical image segmentation [12], [13], [14], [15], [16], [17], [18], [19]. Segmentation methods based on CNNs, Transformers, and Mamba frameworks have shown superior performance across diverse tumor and organ segmentation tasks [20], [21], [22], [23], [24], substantially enhancing accuracy and robustness while significantly reducing radiologists’ workload in clinical auxiliary diagnosis. Although lymph node segmentation approches have been employed in the investigation of different diseases such as cervical cancer, lung cancer, and head-and-neck cancers [25], [26], [27], studies specifically focusing on the segmentation of colorectal cancer lymph node metastasis remain limited. Compared with solid tumors or organ structures, colorectal lymph node metastasis often reside in more complex anatomical surroundings, are susceptible to signal interference, and exhibit small volumes, irregular shapes, and indistinct boundaries, making them difficult to distinguish from postoperative residual lesions [28]. These factors contribute to the suboptimal performance of existing models in handling small-volume structures and blurred boundaries, leading to an increased susceptibility to missed detections, particularly within the complex perirectal anatomical structures. Therefore, researching segmentation methods for CRC LNM based deep learning not only fills a critical research gap but also holds significant potential for improving diagnostic reliability and supporting clinical decision-making.

Another major factor limiting the clinical adoption of automatic segmentation models is their interpretability. Interpretability is one of the key criteria for clinical deployment of computer-aided diagnostic systems. Due to the black-box nature leads to their internal decision-making process lacks interpretability, which significantly reduces their trustworthiness and adoption rate in clinical settings [29]. Consequently, if model outputs cannot be interpreted or validated by clinicians, they are unlikely to be used for decision support. To overcome this barrier, there is an urgent need to develop algorithms with improved interpretability, thereby enhancing traceability and reliability in clinical workflows [30]. Because lymph-node assessment directly affects the extent of surgery, lack of interpretability in the results of the segmentation method will constrain its application in the corresponding clinical scenario. Consequently, improving the method’s transparency and interpretability can not only increase the credibility of the output for physicians but also furnish clinical auxiliary diagnostic systems with a verifiable and reliable foundation for decision-making.

To address these challenges, a new segmentation method UCKAN (U-Convolutional KANs Attention Network) is introduced. The model adopts a U-shaped architecture similar to U-Net. Conventional convolutional layers are replaced with Convolutional Kolmogorov-Arnold Networks (Convolutional KANs) [31], 32], to enhance interpretability. KANs substitute traditional high-dimensional weight matrices with learnable one-dimensional functions, providing a more transparent and interpretable nonlinear mapping mechanism. We further introduce a Global Spatial Multi-Scale Attention (GSMA) module that extracts and fuses global contextual information with multi-scale spatial features, emphasizing both global and local semantic cues. This mechanism enhances the model’s sensitivity to small, low-contrast lymph-node regions, enabling more accurate discrimination between lesions and surrounding tissues. With these designs, UCKAN simultaneously improves segmentation accuracy and interpretability for colorectal cancer lymph-node metastases, offering an effective solution for clinical decision support in anatomically complex settings.

The main contributions of the method demonstrated in this work are outlined below:

1. A novel segmentation method that integrates Convolutional KANs with a Global Spatial Multi-Scale Attention mechanism, specifically designed for segmenting lymph-node metastases in colorectal cancer.
2. We incorporate interpretable Convolutional KANs in place of conventional CNN layers. By using learnable one-dimensional functions instead of high-dimensional convolutional weights, the model provides more transparent and interpretable feature mappings, thereby improving its clinical applicability.
3. We introduce a Global Spatial Multi-Scale Attention module that enhances the detection of small, low-contrast lymph nodes, substantially improving segmentation performance for tiny lesions with indistinct boundaries.

## Related works

### U-shaped network in medical image

Because the U-Net [33] is better than other methods, resulting in U-shaped network has become a dominant framework within the field of medical image segmentation. It applies the framework consisting of an encoder–decoder design with skip connections, enabling efficient feature propagation from downsampling to upsampling for generating segmentation masks. Due to the advantages of the U-Net method, such as low demand for training data and fast training speed, in the area of medical image segmentation, it has received much attention and been widely applied in the field. Hence, amount of U-shaped models by integrating various advanced methods at present have been derived.

Suzuki et al. utilized a normal U-Net [34] for lymph nodes segmentation in gastrointestinal surgery, demonstrating its feasibility in clinical applications. Qiu et al. proposed ERU-Net [35] for uterine lymph node segmentation by embedding ECA attention and residual learning in U-Net. This design effectively addresses the challenges of uneven intensity distributions, blurred boundaries, high background noise, and low contrast in MRI, thereby enhancing feature extraction performance. Manjunatha et al. applied a residual-enhanced U-Net variant [36] to segment mediastinal and abdominal lymph nodes, obtaining satisfactory results. Zhou et al. introduced DFA-UNet [37], built upon ConvNeXt to extract local features and Vision Transformer extract global features for lung cancer lymph node segmentation. Experimental results demonstrated strong segmentation performance. The emergence of Mamba [38] has provided a more advanced solution for medical image segmentation. Similar to Transformers, Mamba is capable of global feature modeling; however, it achieves long-range dependency modeling through a state-space update mechanism with linear complexity (O(n)). This property makes it particularly suitable for capturing long-distance structural relationships in medical images and enhances sensitivity to lesion shape variations and boundary details. Consequently, numerous segmentation methods combining U-Net with Mamba have emerged in medical imaging research [39], [40], [41], [42], [43], [44].

These advanced U-Net–based methods, incorporating residual learning, attention mechanisms, Transformers, and Mamba architectures, have yielded promising results in medical image segmentation. However, from a clinical perspective, research specifically targeting the segmentation of colorectal cancer lymph node metastasis remains limited, creating an urgent demand for automated solutions in clinical workflows. Furthermore, from a methodological standpoint, the core convolutional layers in these models still rely heavily on fixed nonlinear activation functions such as ReLU, which constrains their upper bound for nonlinear feature fitting.

### KAN in medical image segmentation

Since its introduction, KAN [31] has attracted substantial attention in the research community. Compared with multilayer perceptrons (MLPs), KAN offers higher expressive capacity, reduced susceptibility to overfitting, and improved interpretability. A key property of KAN is its ability to represent arbitrary multivariate functions through the additive combination of learnable one-dimensional nonlinear functions. Contrast to MLPs – where nonlinearities such as ReLU are fixed – KAN employs learnable nonlinear functions, thereby providing superior nonlinear approximation capability. Consequently, KAN has been increasingly adopted in the field of various diseases medical image segmentation tasks [45], [46], [47].

Li et al. integrated KAN with U-Net to propose the U-KAN method [46]. This approach incorporates KAN blocks into the convolutional layers of U-Net, enhancing its accuracy, effectiveness, and interpretability. An Innovative interpretable method FunKAN [45] was propesed by Penkin et al. for image processing. They also developed a new segmentation method, U-FunKAN. Experimental results demonstrate that this method achieves strong segmentation performance. Yang et al. proposed MedKAN [47], which comprises a local and global feature extraction module based on KAN to capture complex texture details and contextual features in medical images. Experiments show that MedKAN outperforms CNN- and Transformer-based methods.

However, in image segmentation tasks, KAN struggles to effectively model pixel-level neighborhood relationships, limiting its ability to capture local dependencies in images [32]. To address this limitation, Bodner et al. proposed Convolutional Kolmogorov-Arnold Networks [32], enabling effective capture of local dependencies in images.

## Methods

### Overall method structure diagram

**Figure 1:**
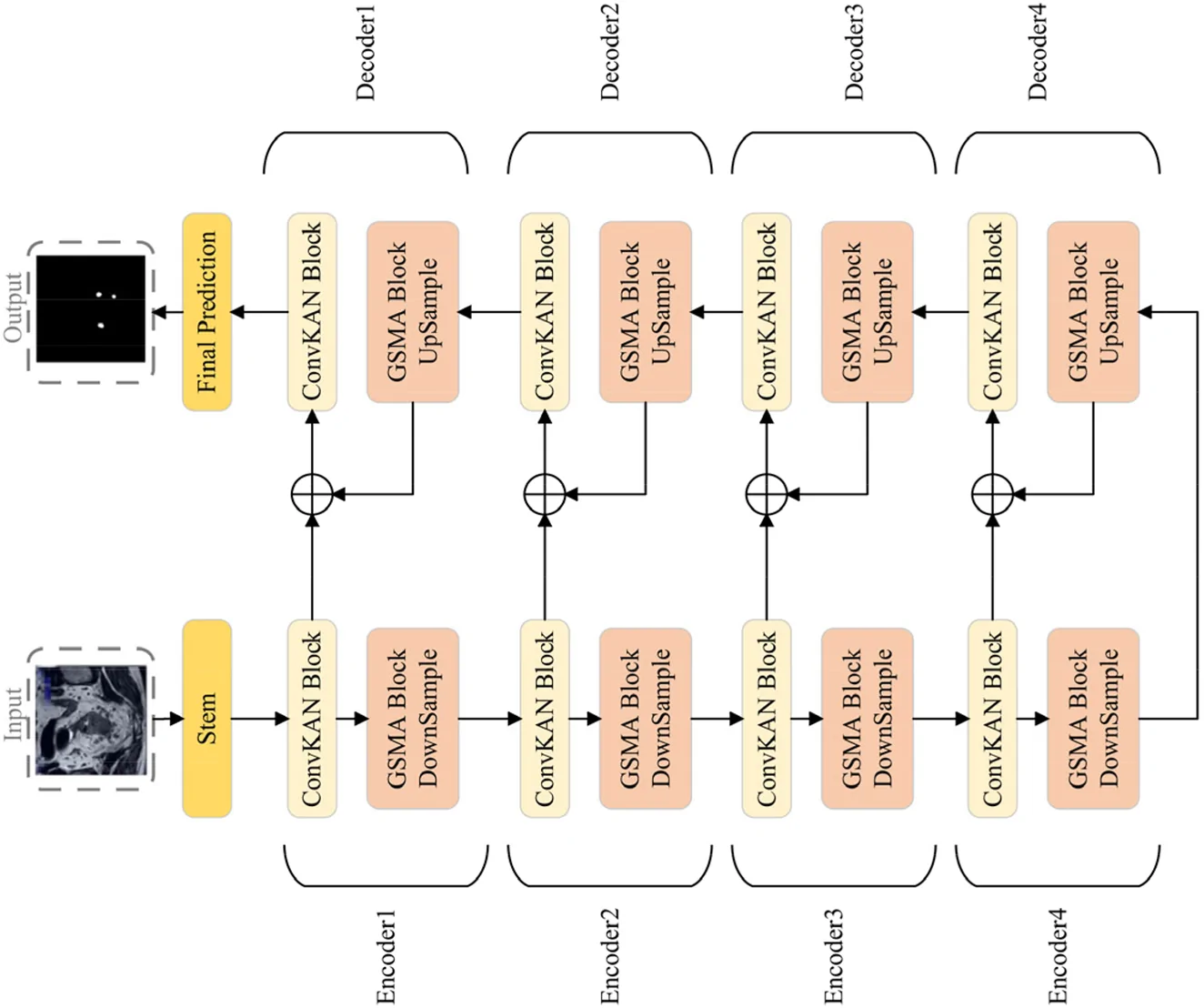
Overall method structure diagram.

Figure 1 shows UConvKAN-AttentionNet (UKCAN) structure diagram, including a two-stage encoder-decoder design and incorporates ConvKAN Blocks, downsampling GSMA Blocks, and upsampling GSMA Blocks. According pipline, the input images are sent to the ConvKAN Blocks and downsampling GSMA Blocks in the encoder, then the feature map flows in the upsampling GSMA Blocks and ConvKAN Blocks in the decoder. Additionally, in order to preserve spatial feature information, the structure that establishes skip connection between the encoder and decoder.

### UKCAN architecture

#### ConvKAN block

**Figure 2:**
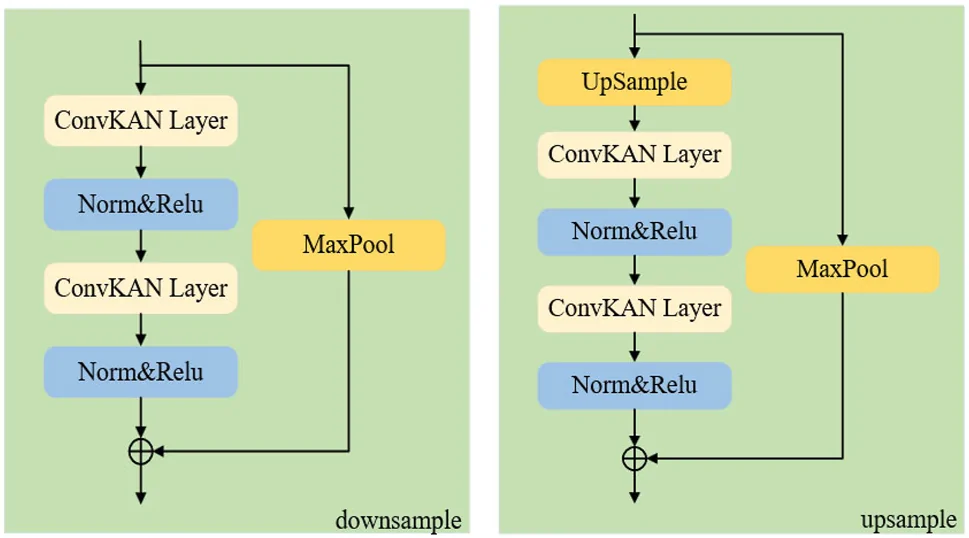
ConvKAN block module.

Figure 2 illustrates both the downsampling ConvKAN Block and the upsampling ConvKAN Block. Each ConvKAN Block consists of three components: two ConvKAN layers, two normalization and ReLU layers, and a skip connection with a MaxPool layer. Each ConvKAN layer uses a 3 × 3 kernel with a stride of 1 and padding of 1, algorithm descriptio is shown in Table1.

**Table 1:** ConvKAN algorithm.

Input: Feature map *X* with shape (*C*_in, *H*, *W*)
Output: Enhanced feature map *Y*with shape (*C*_out, *H*, *W*)
1. Initialize learnable base weights *W*_*b* and spline weights *W*_*s* 2. Set grids points *G* for B-spline functions 3. Compute base linear projection: *X*_base=Conv2*D*(*X*, Kernel_size=*k*, weight=*W*_*b*) 4. Compute spline transformation: a. Expand *X* into spline basis: *B*=Evaaluate_*B*Spline(*X*, *G*) b. Apply learnable weights: *X*_spline=Conv2*D*(*B*, Kernel_size=1, weights=*W*_*s*) 5. Feature fusion: *Y*=Activation(*X*_base+*X*_spline) 6. Return *Y*

Compared with traditional convolutional neural networks, ConvKAN does not adopt the approach that relies on linear weight matrices and fixed activation functions. Instead, based on the Kolmogorov-Arnold representation theorem, the learnable nonlinear activation functions are directly placed on each connection edge of the convolution kernel. This means that the convolution kernel is no longer composed of scalar weights, but consists of a set of learnable unary functions. ConvKAN models the local receptive field as a collection of learnable single-variable splines. This transformation enables the network to adaptively learn the optimal activation response for specific MRI intensity ranges, effectively addressing the challenge of blurred boundaries in microlymph node segmentation. The encoder incorporates MaxPool layers for downsampling, using a 2 × 2 pooling window to retain the most salient features, while the decoder employs bilinear interpolation for upsampling to enlarge feature maps. Formally, given an input image $X_{0}=I\in R^{H_{0}*W_{0}*C_{0}}$ , the output formula of each KAN Block is demonstrated as follows:

(1) $$Y_{\text{down}-l}=\Psi \left(K_{2}\left(\Psi \left(K_{1}\left(X_{l-1}\right)\right)\right)\right)+\text{MaxPool}\left(X_{l-1}\right)$$

(2) $$Y_{up-l}=\Psi \left(K_{2}\left(\Psi \left(K_{1}\left(\text{Bilinear}\left(X_{l-1}\right)\right)\right)\right)\right)+\text{Bilinear}\left(X_{l-1}\right)$$

Here, *K* denotes the ConvKAN operation, Ψ represents the Norm & ReLU operation, + indicates element-wise addition, *Y*_down-l_ and *Y*_up-l_ correspond to the output features of the downsampling and upsampling *l* layers, respectively.

#### GSMA Block

**Figure 3:**
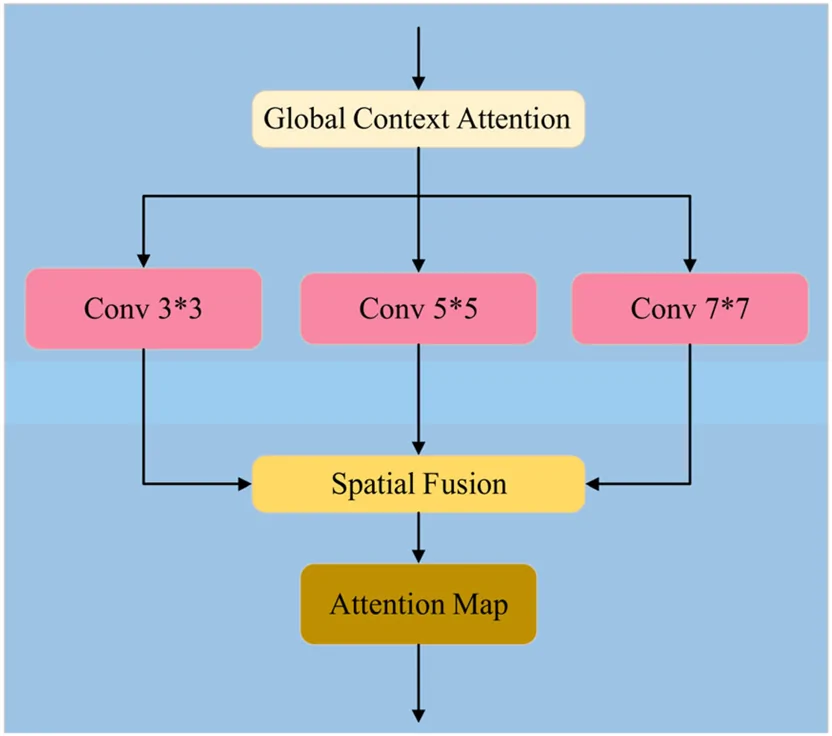
GSMA block module. The GSMA module leverages a parallel branch structure to balance global context and local spatial resolution, effectively calibrating noisy pelvic backgrounds in MRI scans.

Figure 3 illustrates the workflow of the GSMA Block, which integrates global context attention, a multi-scale convolutional networks, and a spatial fusion enhancement module. Table 2 is describe GSMA algorithm. In the MRI images of colorectal cancer lymph node metastatic foci usually have the characteristics of blurred boundaries and complex background anatomical structures (such as interference from blood vessels and fat). Traditional attention mechanisms (such as SE or CBAM) often adopt a single-channel focus or simple serial superposition, which is prone to losing fine spatial geometric features during processing. The GSMA module is designed as a parallel hybrid architecture, aiming to simultaneously capture global context dependencies and fine spatial edge information. Through dynamic calibration of global features on spatial features, it suppresses noise in non-tumor areas. This mixed sensing capability has significant advantages in capturing the characteristics of tiny lymph nodes within a 3–5 mm range in MRI. Formally, *X* as input data is sent into the global context attention module, to produce the refined feature map *X* ^′^, as shown in Equation (3).

(3) $$X^{\prime }=A_{\text{GC}}\left(X\right)$$

**Table 2:** Global spatial multi-scale attention algorithm.

Input: Input feature map *X* from ConvKAN Block
Output: Attention map *M*_Att_
1. Global context Attntion: *X* ^′^=*A*_GC_(*X*) 2. Muti-Scale feature extraction: $C_{3}=Conv2D\left(X^{\prime },\;\ker nel\_size=3,padding=1\right)$ $C_{5}=Conv2D\left(X^{\prime },\;\ker nel\_size=5,padding=2\right)$ $C_{7}=Conv2D\left(X^{\prime },\;\ker nel\_size=7,padding=3\right)$ 3. Spatial attention generation fusion: *F*_concat=Concatenate([*C*_3_, *C*_5_, *C*_7_], axis=chnnnels) *F*=*A*_*sp*_(*F*_concat) 4. Attention feature: *M*_Att_=Attention(*F*) 5. Return *M*_Att_

Then, *X* ^′^ is fed in parallel into three convolutional branches of different kernel sizes for feature extraction, producing feature maps *C*_3_, *C*_5_, *C*_7_, as shown in Equation (4).

(4) $$\left\{\begin{array}{l} C_{3}=F_{3\times 3}\left(X^{\prime }\right)\\ C_{5}=F_{5\times 5}\left(X^{\prime }\right)\\ C_{7}=F_{7\times 7}\left(X^{\prime }\right) \end{array}\right.$$

Here, *C*_3_, *C*_5_, *C*_7_ correspond to 3 × 3, 5 × 5, 7 × 7 the output feature maps of the three convolutions, respectively. These are then fused spatially to produce a new feature map, as expressed in Equation (5).

(5) $$M_{\text{Att}}=\text{Concat}\left(C_{3},C_{5},C_{7}\right)$$

Finally, the output formula of GSMA Block is demonstrated as follows:

(6) $$M_{\text{Att}}=\underset{k\in \left\{3,5,7\right\}}{\sum }F_{k\times k}\left(A_{GC}\left(X\right)\right)$$

## Experiments

### Dataset description

**Figure 4:**
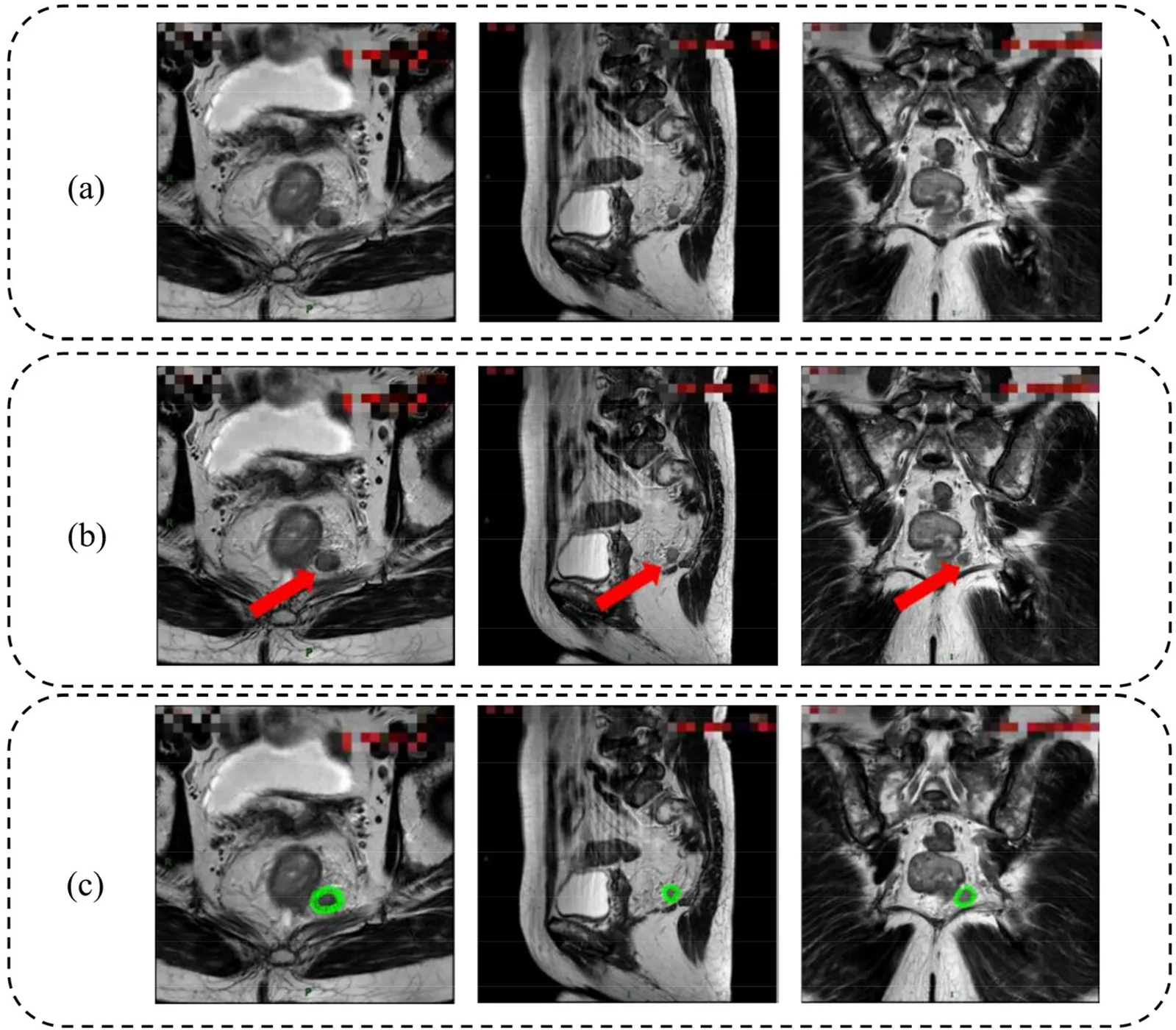
MRI dataset of colorectal cancer lymph node metastasis (CRC LNM) (a) original T2WI scans across sagittal, transverse, and coronal planes. (b) Clinician-identified locations (red arrows) of tiny lesions. (c) Corresponding expert-annotated ground truth masks used for training.

In this study, the data were obtained from a tertiary hospital in Heilongjiang Province, China (Harbin Medical University Cancer Hospital). All patients were examined using a 3.0-T Philips Achieva MRI system equipped with a 16-channel body array coil. Sagittal T2-weighted imaging (T2WI) was first performed with a repetition time/echo time (TR/TE) of 3,000/100 ms, a number of signal averages (NSA) of 2, a slice thickness of 4.0 mm, and an inter-slice gap of 0.4 mm. Based on the sagittal localization of colorectal lesions, axial T2WI scans were subsequently acquired perpendicular to the intestinal lumen, using parameters of TR/TE 3824/110 ms, NSA ranging from 0 to 3, a slice thickness of 3.5 mm, and an inter-slice interval of 0.2 mm. For cases exhibiting lesion orientations parallel to the intestinal axis, additional coronal T2WI scans were obtained with TR/TE of 3,824/110 ms, NSA of 3, and an inter-slice spacing of 0.2 mm. Finally, the target lymph nodes were identified on sagittal, axial, and coronal images. During scanning, patients were required to lie supine with their faces upward, and the scan proceeded from the head to the pelvis. The pelvic region was fully encompassed by the coil, using the horizontal line through the anterior superior iliac spines as a reference for positioning. Target lymph nodes were located on both axial and coronal images. All MRI data were confirmed and cross-checked by radiologists against pathological gold-standard images to ensure the accuracy of the original image properties in this study. Radiologists annotated the images using the LabelMe tool, producing data in the standard VOC dataset format to ensure precise delineation of regions of interest and corresponding annotations. Detailed data information is shown in Figure 4.

The original dataset comprised 275 cases. Then the dataset is divided into training, validation and testing sets in a ratio of 8:1:1 to ensure the uniqueness of the data. After data augmentation using geometric transformations such as vertical and horizontal flips, the dataset was expanded to 1,100 samples. Then, each sample was then cropped to a size 256 × 256 of and normalized.

### Evaluation metrics

The proposed UCKAN method will be evaluated in colorectal cancer lymph node metastasis segmentation, both overlap-based metrics and boundary distance metrics were employed in the experiments. First, the Dice Similarity Coefficient (DSC) and Intersection over Union (IoU) were used to quantify the degree of overlap between the predicted and ground-truth regions. They are defined as follows:

(7) $$\text{Dice}=\frac{2\left|P\cap G\right|}{\left|P\right|+\left|G\right|}$$

(8) $$\text{IoU}=\frac{\left|P\cap G\right|}{\left|P\cup G\right|}$$

Here, *P* and *G* represent the model-predicted mask and the ground-truth mask, respectively.

Next, Precision and Recall metrics were introduced, these were used to demonstrate the ability of the model to classify the nature of CRC LN. The formulas are listed:

(9) $$\mathrm{Pr}\;e\text{cision}=\frac{\text{TP}}{\text{TP}+\text{FP}}$$

(10) $$\text{Recall}=\frac{\text{TP}}{\text{TP}+\text{FN}}$$

Here, the abbreviations TP, FP FN indicate the numbers of true positives, false positives, and false negatives, respectively.

Third, owing to the small size and unclear boundaries of CRC LN, relying solely on overlap-based metrics is insufficient to fully assess the model’s boundary fitting capability. Therefore, this study further employed the 95 % Hausdorff Distance (HD95) as a boundary distance metric. HD95 is defined as the 95th percentile of distances between the predicted and ground-truth boundaries, which reduces the impact of extreme noise points. The formula is presented as follows:

(11) $$\text{HD}95=\text{percentil}e_{95}\left(d\left(P,G\right)\right)$$

*d*=(·) represents the minimum Euclidean distance from a point to a set. Using the aforementioned evaluation metrics, a comprehensive assessment of the performance of the UCKAN method was conducted.

### Implementation details

To ensure the consistency of the experimental results, all the experiments were conducted under the same conditions. The PyTorch as deep learning framework was used in all experiments. Ubuntu 22.04 and A100 with 80 GB of memory were selected as the operating system and GPU for all the experimental environments. Other parameters were trained using the stochastic gradient descent (SGD) algorithm. The training parameters were set as follows: a learning rate of 0.001, momentum of 0.9, 200 training epochs, and a batch size of 64.

### Performance comparsion

This study conducted benchmark evaluations of the proposed UCKAN method, including both ablation studies and comparative experiments. In the ablation studies, each part of the experiment were designed to show the effectiveness of each core component and to assess their contributions to colorectal cancer lymph node metastasis segmentation. Table 1 described the final experimental results. To ensure comparability, all experimental strategies remain consistent.

**Figure 5:**
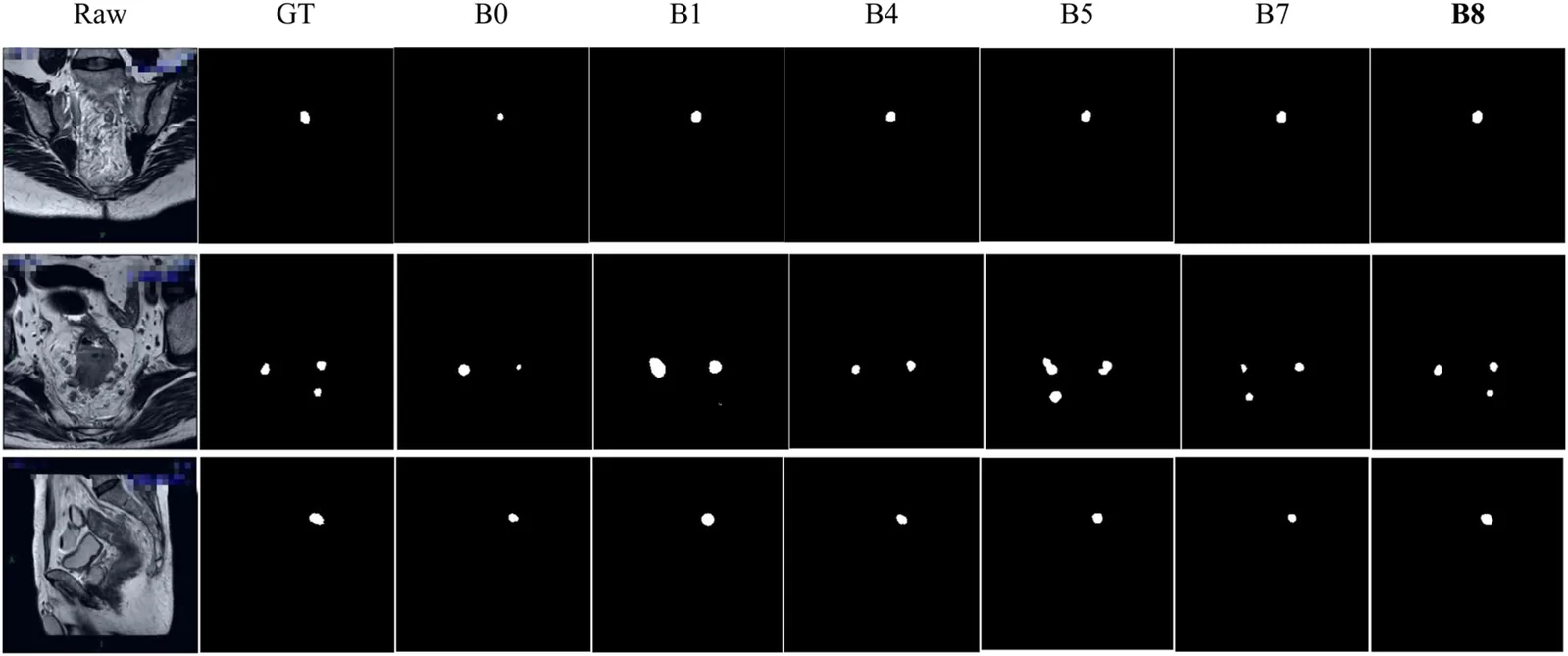
Visualization of segmentation results.

The comparative experiment could fully demonstrate the performance of UCKAN. Therefore, UCKAN compared it with the other eight methods: U-Net, Seg_UKAN, AttU_Net, R2U_Net, R2AttU-Net, NestedUNet, TransformerUNet, MambaUNet. These comparative methods encompass a variety of network architectures, including CNNs, attention mechanisms, Transformers, Mamba, and KAN, providing a comprehensive validation of the proposed method across diverse classic network frameworks. The quantitative results of UCKAN compared with these methods are presented in Table 2, while selected visualizations are shown in Figure 5.

#### Ablation experiment

Based on the ablation experiments, the influence of each component on the model during feature extraction was evaluated, with the detailed experimental design summarized in Table 3. In experiment A0, the Dice, IoU, and Recall scores were 89.88 , 81.78, and 88.34 %, respectively. In experiment A4, replacing the convolutional layers in U-Net with ConvKAN resulted in improvements of 0.43 , 0.79, and 0.74 % in Dice, IoU, and Recall, respectively. The results indicate that ConvKAN, with its learnable nonlinear activation functions, has a stronger nonlinear fitting capability, enabling more effective feature extraction from colorectal cancer lymph node metastasis samples. In experiments A0, A1, A3, and A4, incorporating the GSMA module led to segmentation metrics exceeding those of U-Net. This suggests that the GSMA module, by integrating global context and multi-scale information, effectively enhances lesion saliency and optimizes multi-scale feature fusion. Among experiments A0–A7, A7 is the best, with Dice, IoU, and Recall scores of 91.52 , 83.29, and 90.78 %, surpassing any individual improvement in experiments A0–A6. These results demonstrate that ConvKAN and the GSMA module exhibit strong synergy in analyzing features of colorectal cancer lymph node samples.

**Table 3:** Ablation experiments of the proposed UCKAN method.

ID	ConvKAN	Encoder GSMA	Decoder GSMA	Dice↑	IoU↑	Recall↑
A0	**×**	**×**	**×**	89.88	81.78	88.34
A1		**√**	**×**	90.17	82.37	88.84
A2		**×**	**√**	90.25	82.52	88.69
A3		**√**	**√**	90.34	82.64	89.31
A4	**√**	**×**	**×**	90.31	82.57	89.08
A5		**√**	**×**	90.47	82.64	89.67
A6		**×**	**√**	91.11	83.07	90.16
**A7**		**√**	**√**	**91.52**	**83.29**	**90.78**

In the table, ↑ indicates that the higher the value, the better. The bold values represent the best results.

#### Comparative experiment

In the comparative experiments, the quantitative metrics performance of UCKAN has achieved the strongest, all experimental results are shown in Table 4. UCKAN achieved Dice, IoU, Precision, Recall, and HD95 scores of 91.52 , 83.29, 93.25, 90.78 %, and 1.93, respectively. These values surpass the second-best model, MambaUNet, by 0.68 , 1.56, 2.01, 1.47 %, and −0.2 in the respective metrics. The results indicate that UCKAN possesses strong feature extraction capabilities, benefiting from ConvKAN’s nonlinear fitting ability and the GSMA module’s extraction of global context and multi-scale features. Notably, the HD95 performance of UCKAN is better than other methods, describing its ability to accurately delineate lesion boundaries, thereby facilitating reliable clinical assessment of colorectal cancer lymph node metastasis.

**Table 4:** Comparative experiments of the proposed UCKAN method.

ID	Model	Dice↑	IoU↑	Precision↑	Recall↑	HD95↓
B0	U-Net	89.88	81.78	91.71	88.34	4.48
B1	Seg_UKAN	89.98	81.93	90.66	89.41	4.26
B2	AttU_Net	89.66	81.44	90.91	86.68	4.11
B3	R2U_Net	89.61	81.34	90.85	86.73	4.17
B4	R2AttU_Net	89.86	81.75	90.56	88.96	2.83
B5	NestedUNet	90.34	82.64	91.57	87.62	2.79
B6	TransformerUNet	90.06	81.11	90.53	87.61	2.05
B7	MambaUNet	90.84	81.73	91.24	89.31	2.13
**B8**	**UCKAN(Ours)**	**91.52**	**83.29**	**93.25**	**90.78**	**1.93**

In the table, ↑ indicates that the higher the value, the better, ↓ indicates that the lower the value, the better. The bold values represent the best results.

To more intuitively demonstrate UCKAN’s superior performance on the colorrectal cancer lymph node metastasis dataset, segmentation visualizations were generated for the methods corresponding to experiment IDs B0, B1, B4, B5, B7, and B8 in Table 4. Three challenging categories of test samples were selected for qualitative comparison: (a) tiny lesions, (b) multiple lesions, and (c) lesions with very low contrast against surrounding tissues. The visualization results are presented in Figure 5.

As shown in Figure 5(a), for tiny lymph nodes, U-Net exhibits noticeable incomplete segmentation, and other methods fail to delineate the lymph node boundaries. In contrast, UCKAN clearly segments the edges of the lymph nodes, outperforming the other methods. This improvement is primarily attributed to the stronger feature fitting capability of the ConvKAN units, which prevents the loss of fine details in deep network processing.

As shown in Figure 5(b), U-Net, Seg_UKAN, and R2AttU-Net fail to segment all lesions, indicating deficiencies in extracting multi-target features. Although NestedUNet can identify multiple targets, noticeable over-segmentation occurs at the lesion boundaries. MambaUNet produces the correct number of segmented lesions matching the ground truth, but the lesion sizes are inconsistent, resulting in under-segmentation. In contrast, UCKAN, leveraging the GSMA module’s global context and multi-scale feature extraction capabilities, accurately determines lesion locations and edges. Its segmentation results are both quantitatively and qualitatively precise and smooth, showing a smaller gap compared to the ground truth.

Figure 5(c) demonstrates the lesions are located in a complex environment with low contrast against surrounding tissues. All methods exhibit deviations in segmentation details; however, UCKAN, leveraging ConvKAN’s nonlinear fitting ability and the GSMA module’s extraction and spatial fusion of global multi-scale features, more effectively highlights salient lesion features, achieving clearer and more accurate segmentation.

B0–B8 correspond to the experiment IDs in Table 2. Raw indicates the original samples. GT denotes the ground truth, and B0-B8 show visualized the segmentation results from the respective experiments.

In summary, Table 4 and Figure 5 describe the results of the segmentation method indicators and the visualized segmentation results consistently demonstrate that UCKAN outperforms other segmentation methods. UCKAN effectively leverages ConvKAN to increase feature representation capability and employs the GSMA module to precisely guide feature focus, enabling it to achieve maximal accuracy while efficiently addressing common challenges in clinical image segmentation.

**Figure 6:**
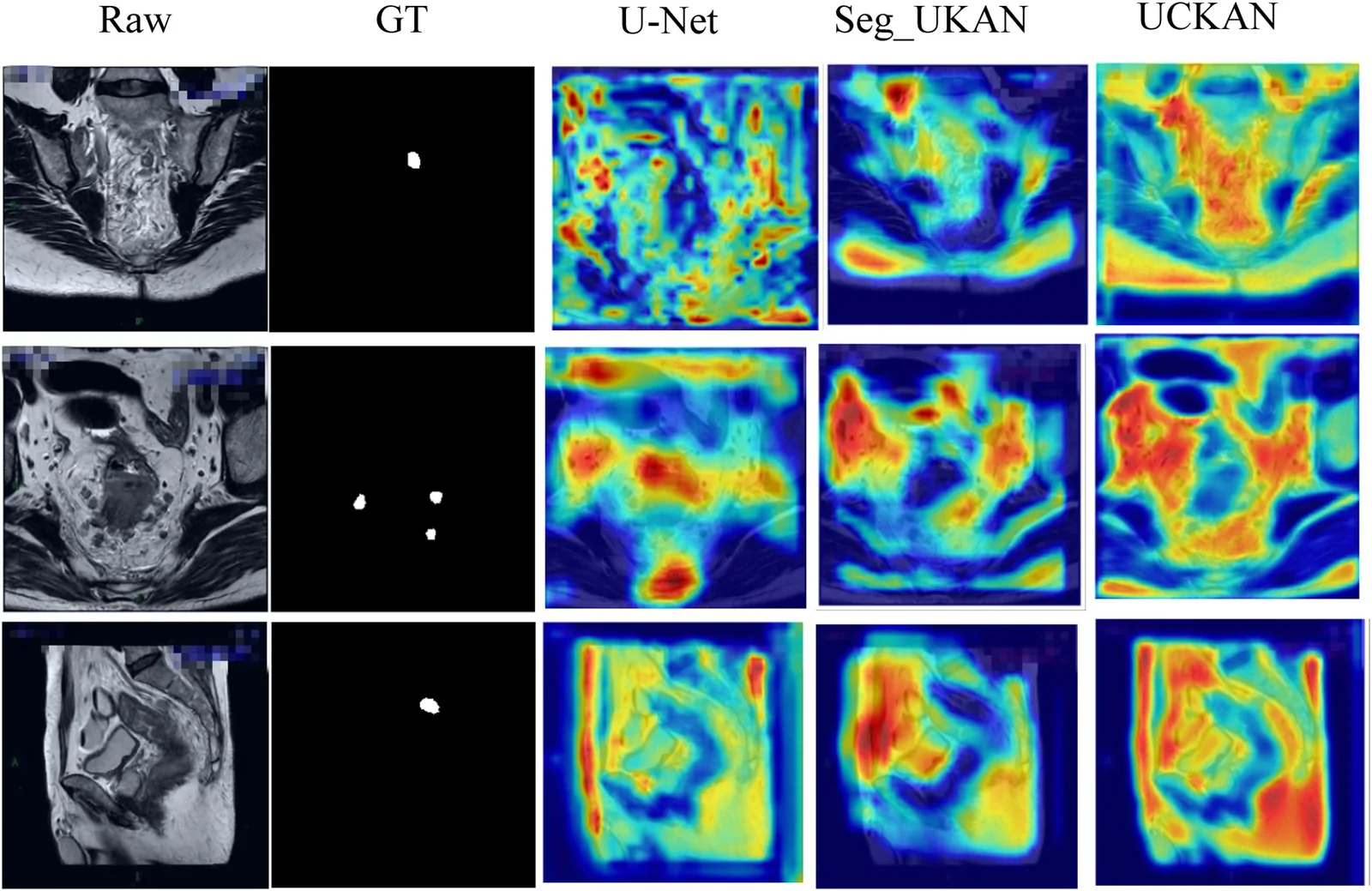
UCKAN result heatmap.

As shown in Figure 6, the UCKAN proposed by us demonstrated a more precise and focused activation effect in actual lymph node lesions. This is attributed to the ConvKAN part, which can learn nonlinear basis functions that are highly sensitive to the subtle texture gradients of cancer tissues, as well as the GSMA module, which can suppress irrelevant background noise. This intuitive evidence confirms that the outstanding performance of UCKAN stems from its ability to understand and locate relevant clinical features, providing a highly reliable second opinion for radiologists, thereby enhancing its reliability in clinical decision support and providing a basis for preoperative personalized quality planning.

#### Statistical validation experiment

To further verify the generalization ability and statistical stability of UCKAN, we conducted strict five-fold cross-validation and statistical analysis. The dataset was divided into five mutually exclusive subsets at the patient level to prevent data leakage. As shown in Table 5, the average Dice score of UCKAN reached 91.48 %, with a standard deviation of only±0.05 %. The results indicate that this architecture shows high consistency and robustness under different data partitions, and is not the result of a specific random partition. This stability proves the reliable potential of UCKAN in the clinical application of rectal cancer lymph node metastasis segmentation. As shown in Table 6, all p-values are less than 0.05, confirming that the performance improvement of UCKAN is statistically significant and not caused by random accidental factors.

**Table 5:** UCKAN 5-fold cross-validation.

Fold	Dice↑	IoU↑	Precision↑	Recall↑	HD95↓
Fold1	91.48	83.45	93.16	90.82	1.91
Fold2	91.52	83.32	93.35	90.76	1.96
Fold3	91.44	83.31	93.14	90.63	1.89
Fold4	91.41	83.27	93.22	90.77	1.95
Fold5	91.52	83.29	93.25	90.78	1.93
Average	91.48	83.45	93.16	90.82	1.91
Std. Dev.	±0.05	±0.07	±0.08	±0.07	±0.03

In the table, ↑ indicates that the higher the value, the better, ↓ indicates that the lower the value, the better.

**Table 6:** Statistical significance (p-values) of UCKAN vs. baseline models.

Comparison par	Metric	*t*-statistic	p-Value	Significance
UCKAN vs. U-Net	Dice	6.48	<0.001	^c^
UCKAN vs. Seg-UKAN	Dice	4.33	0.0015	^b^

^a^p<0.5 (significant), ^b^p<0.01(highly significant), ^c^p<0.001(extremely significant).

## Disscussion

The proposed UCKAN method achieved satisfactory results in colorectal cancer lymph node metastasis segmentation, with Dice, IoU, Precision, Recall, and HD95 scores of 91.52 , 83.29, 93.25, 90.78 %, and 1.93, respectively. This satisfactory result is achieved through the synergistic between the ConvKAN and GSMA modules. Traditional convolutional layers typically rely on fixed nonlinear activation functions (e.g., ReLU), which limits their expressive capacity. In contrast, ConvKAN benefits from the learnable nonlinear mechanism introduced by the KAN network, allowing convolutional kernels to perform flexible nonlinear mappings through functions such as B-splines. This enhances adaptive capability and performance during fine feature extraction, facilitate the modeling of low-contrast lesions and complex edge features by the network. The proposed GSMA module provides comprehensive analysis of colorectal cancer lymph node metastasis features across global context and multi-scale dimensions, effectively addressing the limited local information of small lymph nodes and enhancing feature discriminability.

The results of the segmentation indicators for all the experimental methods are shown in Table 4, including Dice, IoU, and Recall. The performance of UCKAN is the best in these methods. Although TransformerUNet and MambaUNet have advantages in global feature extraction, they are limited by global average pooling when handling precise local boundaries. Through the GSMA module, UCKAN not only enhances the extraction of global features, but also has significant advantages in the extraction of local features. Compared with methods like Seg_UKAN that rely on a single structural innovation, UCKAN adopts multi-level composite innovation, which can extract richer semantic features and thus ensure higher accuracy of the final segmentation results.

The results of the statistical validation analysis are presented in Tables 5 and 6. The extremely low standard deviation (Dice: 91.48 % ± 0.05 %) demonstrated by 5-fold cross-validation has significant clinical reference value. In clinical practice, this means that UCKAN exhibits excellent diagnostic consistency. Regardless of any anatomical variations or different MRI scanning sequences, the model can provide stable and predictable segmentation results. This algorithm’s robustness reduces the risk of misdiagnosis caused by model fluctuations, providing radiologists with a reliable second opinion, and enhancing the clinical trustworthiness of the AI-assisted diagnostic system. Furthermore, Statistical stability and significance are the prerequisites for the clinical application of AI technology. The significant improvement (p<0.001) in multiple indicators and the high consistency across different data subsets of UCKAN directly translate into a more accurate identification ability for micro metastasis of lymph nodes in clinical practice. This has profound implications for the precise staging of rectal cancer patients and the formulation of individualized surgical plans.

By integrating the result heatmaps into the clinical workflow analysis, it has been proven that UCKAN is not merely a segmentation mask; it also provides a transparent decision support tool. The heatmaps confirm that UCKAN can accurately identify the key texture features of metastatic nodules, which enables radiologists to be confident in using the automatic measurement results for preoperative personalized treatment planning. This synergy demonstrates how high-precision and interpretable methods can transform traditional subjective and time-consuming manual workflows into standardized and efficient clinical processes.

The UCKAN method can automatically and accurately delineate metastatic colorectal cancer lymph nodes, providing valuable guidance for preoperative clinical decision-making. It also significantly reduces the time required for manual lesion annotation by clinicians, thereby improving the efficiency of preoperative assessment. However, UCKAN’s performance in fine-grained segmentation of lesion boundaries still requires improvement. In addition, being trained on a single-center dataset, the generalizability of UCKAN needs to be further validated using multi-center and multi-device datasets.

Despite the promising performance, a primary limitation of this study is its single-center design with 275 cases. While rigorous data augmentation was applied to simulate inter-scanner variability, future studies involving diverse datasets from multiple manufacturers (e.g., Siemens, GE) are necessary to further confirm the model’s robustness in broader clinical settings.

In the future, we will focus on integrating Edge-aware Loss functions to further address the boundary ambiguity. Traditional Dice loss often treats all pixels equally, whereas an edge-aware approach applies a distance-based or gradient-based penalty to the boundary regions. For instance, incorporating the Hausdorff Distance (HD) Loss could explicitly minimize the maximum distance between the predicted and ground truth contours, thereby refining the fine-grained segmentation of micro-lymph nodes.

## Conclusions

In this paper, an innovative method UCKAN was introduced for segmenting metastatic colorectal cancer lymph nodes. The core innovation lies in using the ConvKAN module to extract sample features, which alters the traditional convolution-based feature extraction method of the U-Net structure, significantly enhancing the model’s nonlinear fitting ability for complex features. Additionally, GSMA modules are embedded in both the encoder and decoder paths, enabling precise global and multi-scale analysis of lymph node features. Evaluation on real medical imaging tasks demonstrates that the performance of UCKAN achieves superior segmentation results, indicating that the proposed innovations effectively overcome the limitations of CRC LNM images low-contrast and multi-scale lesions. This provides a reliable quantitative basis for preoperative planning of personalized surgical strategies and holds significant potential for clinical translation.

Although UCKAN demonstrates excellent performance, there remains room for improvement in fine-grained segmentation of lesion boundaries. Future work will focus on enhancing UCKAN’s ability to extract and model boundary features, for example by incorporating boundary-aware loss functions or more efficient edge detection mechanisms, aiming to achieve near-perfect clinical-level accuracy. Additionally, the clinical generalizability and robustness of UCKAN need to be further validated on multi-center, multi-device datasets.
